# CircPRELID2 functions as a promoter of renal cell carcinoma through the miR-22-3p/ETV1 cascade

**DOI:** 10.1186/s12894-024-01490-z

**Published:** 2024-05-10

**Authors:** Xi Lin, Yi Zhi

**Affiliations:** Department of Urology, The Third Affiliated Hospital of Chongqing Medical University, No.1 Shuanghu Branch Road, Huixing Street, Yubei District, Chongqing City, 401120 PR China

**Keywords:** RCC, circPRELID2, miR-22-3p, ETV1 expression, Malignant progression

## Abstract

**Background:**

Emerging evidence has indicated that a number of circular RNAs (circRNAs) participate in renal cell carcinoma (RCC) carcinogenesis. Nevertheless, the activity and molecular process of circPRELID2 (hsa_circ_0006528) in RCC progression remain unknown.

**Methods:**

CircPRELID2, miR-22-3p and ETS variant 1 (ETV1) levels were gauged by qRT-PCR. Effect of the circPRELID2/miR-22-3p/ETV1 axis was evaluated by detecting cell growth, motility, and invasion. Immunoblotting assessed related protein levels. The relationships of circPRELID2/miR-22-3p and miR-22-3p/ETV1 were confirmed by RNA immunoprecipitation (RIP), luciferase reporter or RNA pull-down assay.

**Results:**

CircPRELID2 was up-regulated in RCC. CircPRELID2 silencing suppressed RCC cell growth, motility and invasion. Moreover, circPRELID2 silencing weakened M2-type macrophage polarization in THP1-induced macrophage cells. CircPRELID2 sequestered miR-22-3p, and circPRELID2 increased ETV1 expression through miR-22-3p. Moreover, the inhibitory impact of circPRELID2 silencing on RCC cell malignant behaviors was mediated by the miR-22-3p/ETV1 axis. Furthermore, circPRELID2 knockdown in vivo hampered growth of xenograft tumors.

**Conclusion:**

Our study demonstrates that circPRELID2 silencing can mitigate RCC malignant development through the circPRELID2/miR-22-3p/ETV1 axis, highlighting new therapeutic targets for RCC treatment.

**Supplementary Information:**

The online version contains supplementary material available at 10.1186/s12894-024-01490-z.

## Highlights

(1) CircPRELID2 silencing impacts RCC cell phenotypes.

(2) CircPRELID2 sequesters miR-22-3p.

(3) CircPRELID2 increases ETV1 expression through miR-22-3p.

**Supplementary Information:**

The online version contains supplementary material available at 10.1186/s12894-024-01490-z.

## Introduction

Renal cell carcinoma (RCC) is one of the most aggressive malignancies [1]. In spite of great advances in treatment methods, these patients in metastatic stages still have a poor prognosis [2]. Hence, searching for more effective therapeutic targets for RCC is very urgent. M2-polarized macrophages exert pro-tumorigenic activity in human cancers, including RCC [3, 4]. Inhibition of M2 macrophage polarization has been considered as a potential therapeutic approach against cancer [5]. Therefore, research in suppressing M2 macrophage polarization may provide new strategies for RCC treatment.

Circular RNAs (circRNAs) are covalently closed RNA molecules produced by back-splicing of pre-mRNAs [6, 7]. Emerging evidence has indicated that a number of circRNAs mediate gene expression by binding to microRNAs (miRNAs), highlighting the crucial implications of such interactions in RCC [8, 9]. As an example, hsa_circ_001895 is up-regulated in clear cell RCC, and it enhances the disease development by increasing SOX12 expression by acting as a miR-296-5p sponge [10]. CircRNA_000926 is prominently elevated in RCC, and its silencing suppresses RCC development via the miR-411/cadherin 2 (CDH2) axis [11]. As for circPRELID2 (hsa_circ_0006528), it has been discovered to enhance breast tumorigenesis and resistance development [12–14]. In the preliminary search for oncogenic circRNAs in RCC using bioinformatics, we found six circRNAs. Among these circRNAs, no studies proved the association of circPRELID2 with RCC. We thus wanted to elucidate the function and molecular process of circPRELID2 in RCC development.

Previous evidence has uncovered that miR-22 is down-regulated in RCC, and the overexpression of miR-22 can suppress RCC progression [15, 16]. Moreover, miR-22-3p participates in RCC cell motility and growth by mediating the PI3K/Akt pathway [15]. However, the influence of the circPRELID2/miR-22-3p interaction on RCC progression is still unclear. Two putative complementary sites among circPRELID2, miR-22-3p and ETS variant 1 (ETV1) were predicted by several computational methods. By combining these experiments in vitro and in vivo, this study has led to the identification of circPRELID2 silencing that can exert an anti-tumor function in RCC via the miR-22-3p/ETV1 cascade.

## Materials and methods

### Bioinformatics

The GEO database (https://www.ncbi.nlm.nih.gov/geo/query/acc.cgi?acc=GSE100186) was used to analyze the aberrant circRNAs from 4 pairs of RCC tissues and adjacent normal tissues. The circBank database was employed to predict the miRNAs that potentially bind to circPRELID2. Analyses for the molecular targets of miR-22-3p were carried out using several computational methods miRWalk, TargetScan, StarBase and miRcode.

### Clinical samples and cells

With the protocols approved by The Third Affiliated Hospital of Chongqing Medical University Ethics Committee, 52 patients with RCC (including 29 cases TNM stage I + II and 23 cases III stage, and 30 cases negative lymph node metastasis and 22 cases positive lymph node metastasis) were enrolled from The Third Affiliated Hospital of Chongqing Medical University. Under the supervision of two pathologists, we obtained tumor tissues and matched healthy tissues from these patients. Informed consent was provided by all patients.

Human proximal renal tubular epithelial HK2 cells (ATCC, Rockville, MD, USA) were reproduced in DMEM/F-12 (Life Technologies, Lucerne, Switzerland), and human RCC cell lines A498, ACHN, 769-P and 786-O (ATCC) and RCC4 (Bnbio, Beijing, China) were maintained in 10% FBS RPMI-1640 medium (Life Technologies) plus 1% antibiotics (Life Technologies) in 5% CO_2_ at 37 °C.

### qRT-PCR

The TRIzol (Life Technologies) was applied for RNA preparation. cDNA generation was implemented using the PrimerScript RT kit for circPRELID2 and the indicated human gene mRNAs and the TaqMan MicroRNA RT Kit for miR-22-3p. Then, SYBR Green (TaKaRa) was employed for qRT-PCR. The primers shown in Supplement Table 1 were used for PCR amplification. Fold changes were scored by the 2^−ΔΔCt^ method after normalization by β-actin or U6.

### Constructs, transfection and transduction

Using Lipofectamine 3000 (Life Technologies), RCC4 and 786-O cells were transiently transfected with sh-circPRELID2 vector (Geneseed, Guangzhou, China), OE-ETV1 expression plasmid (Life Technologies), or sh-NC control (Life Technologies). MiR-22-3p change was done using the commercially available mimic of miR-22-3p or the inhibitor of miR-22-3p (anti-miR-22-3p). Sh-circPRELID2 lentiviral particles were obtained from GenePharma, with nontarget virus particles (sh-NC) as the negative controls. The virus particles were then used to infect 786-O cells, and virus-infected cells were selected by puromycin.

### Treatment of RNase R and actinomycin D

For Actinomycin D treatment, RCC4 and 786-O cells were treated with 2 mg/mL of Actinomycin D (Sigma-Aldrich, Zwijndrecht, The Netherlands) for 0, 4, 8, and 12 h. For RNase R treatment, total RNA (10 µg) was subjected to RNase R incubation (Geneseed), and then RNA was purified by the RNA Purification Kit (Invitrogen, Tokyo, Japan). In both assays, the assessment of PRELID2 mRNA and circPRELID2 levels was done by qRT-PCR.

### Subcellular fractionation assay

The Cytoplasmic & Nuclear RNA Purification Kit was utilized to isolate cytoplasmic and nuclear RNA from cytoplasmic and nucleus fractions of RCC4 and 786-O cells, following the recommendations of the producer (Norgen Biotek, Thorold, ON, Canada). The levels of circPRELID2 were evaluated by qRT-PCR.

### Cell proliferation, colony formation and cell cycle assays

On 50–60% confluency, RCC4 and 786-O cells were performed the transfection of sh-NC, sh-circPRELID2, sh-circPRELID2 + anti-miR-NC mimic or sh-circPRELID2 + anti-miR-22-3p mimic. Cell proliferation test was done using CCK-8 assay. As reported previously [17], we performed colony formation experiment by seeding cells into 6-well plates and culturing them for 14 days. After propidium iodide (Sigma-Aldrich) staining, we scored cell cycle distribution by using a FACSCalibur instrument (BD Biosciences, Heidelberg, Germany).

### Assessment of M2-polarized macrophages by flow cytometry

RCC4 and 786-O cells were performed with the different transfection of sh-NC, sh-circPRELID2, sh-circPRELID2 + anti-miR-22-3p mimic or sh-circPRELID2 + OE-ETV1. After 48 h of transfection, the culture media were collected and used to treat THP-1 monocytic leukemia cells, which were co-treated with 100 ng/mL PMA (Sigma-Aldrich) for macrophage (THP1-M0) induction. 24 h later, single-cell suspensions were stained with FITC-labeled CD206 antibody (#321103, Biolegend, USA) and subsequently incubated with PC5.5-labeled 7AAD (Biolegend) for dead cell elimination. The CD206^+^ cells were scored on the FACSCalibur instrument.

### Transwell assays

Cell transfection was done as described above, and the migration and invasion capacities were detected after 24 h transfection using 24-transwell chambers and invasion chambers pre-coated with Matrigel (BD Biosciences), respectively. We plated transfected cells in serum-free medium into the upper chamber. Media containing 10% FBS were placed into the under chamber. 24 h later, the migrated or invaded cells were photographed and counted after staining.

### Animal studies

All animal procedures were performed following an approval of the Animal Care and Use of The Third Affiliated Hospital of Chongqing Medical University. The animal procedures complied with International guidelines. Ten athymic Balb/c female nude mice (Vital River Laboratory, Beijing, China) were used. The xenograft tumors were generated by subcutaneously injecting sh-NC or sh-circPRELID2 lentivirus-transduced H1299 cells (2 × 10^6^ cells per mouse) into the mice. Tumor growth was monitored by determining tumor volume (0.5 × length × width^2^). After 28 days, all mice were euthanized, and followed by the collection of xenograft tumors. Sections of xenograft tumors were subjected to immunohistochemistry (IHC) processing as reported [18] using an antibody against Ki67 (1:500 dilution, ab16667; Abcam).

### Immunoblotting

Preparation of cell lysates and immunoblotting were conducted using standard protocols [17]. Anti-ETV1 (ab136121) and anti-β-actin (ab8226, all from Abcam, Cambridge, UK) antibodies were employed. To ensure a clearer presentation of the target bands, we removed the excess of the protein gels before the membrane transfer. The original blot images were showed in Additional file 2.

### RNA pull-down, luciferase reporter and RNA immunoprecipitation (RIP) assays

The circPRELID2 segment harboring the miR-22-3p-binding sequence and ETV1 3’-UTR were ligated into the pmirGLO vector (Promega, Mannheim, Germany). Via a TaKaRa MutanBEST Kit, we generated site-directed mutations of the two reporter constructs in the seed region. We introduced report constructs into 293 T cells along with mimic of miR-22-3p or miR-NC. Luciferase activities were determined after 48 h.

For RIP experiments, we treated total extractions of RCC4 and 786-O cells with protein A/G beads-linked anti-Ago2 or isotype anti-IgG antibody (Abcam). For RNA pull-down experiments, we probed total extractions with Biotinylated miR-22-3p mimic (Bio-miR-22-3p) or its mutant in the seed sites (Bio-miR-22-3p-MUT), or Bio-miR-NC control and Streptavidin beads (Life Technologies). RNA bound to beads was subjected to detection of circPRELID2 enrichment.

### Statistical analysis

The *P* values were detected by Student’s *t*-test and ANOVA, and *P* < 0.05 meant the statistical significance. Correlations among circPRELID2, miR-22-3p and ETV1 were analyzed by Spearman rank correlation test.

## Results

### Up-regulation of circPRELID2 in human RCC

**Fig. 1:**
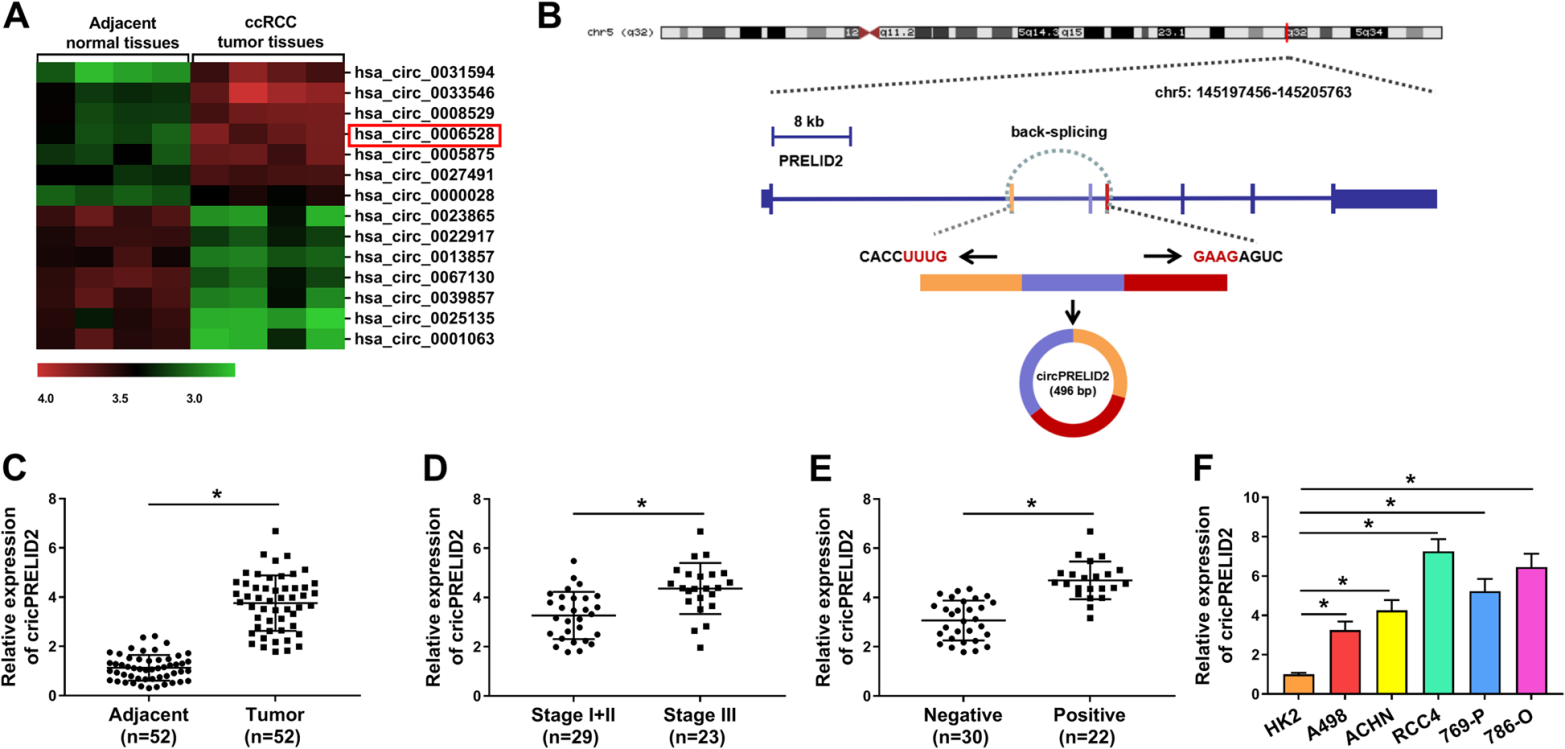
CircPRELID2 expression is increased in RCC tissues and cells. **A** Cluster heat map of dysregulated circRNAs from 4 pairs of RCC tissues and adjacent normal tissues. **B** Schematic diagram of the biogenesis of circPRELID2 via back-splicing mechanism. CircPRELID2 expression by qRT-PCR in 52 pairs of clinical RCC tissues and matched healthy tissues (**C**), 29 cases RCC patients with TNM stage I + II and 23 cases patients with stage III (**D**), 30 cases RCC patients with negative lymph node metastasis and 22 cases patients with positive metastasis (**E**), HK2, A498, ACHN, RCC4, 769-P and 786-O cells (**F**). *P < 0.05

For exploration the related circRNAs in RCC progression, the GEO database was used to observe aberrant circRNAs. CircPRELID2 (hsa_circ_0006528) was highly expressed in RCC tissues (Fig. 1A). The synthesis of circPRELID2 was based on the back-splicing mechanism, and circPRELID2 was generated by exons 2–5 of PRELID2 pre-mRNA (Fig. 1B). CircPRELID2 was prominently augmented in RCC tissues when comparing to the healthy controls (Fig. 1C). Remarkably, the augmentation of circPRELID2 was closely linked to tumor lymph node metastasis and TNM stage (Fig. 1D and E). Similarly, circPRELID2 was present at higher levels in RCC cells (Fig. 1F).

### Characteristics of circPRELID2 in RCC cells

**Fig. 2:**
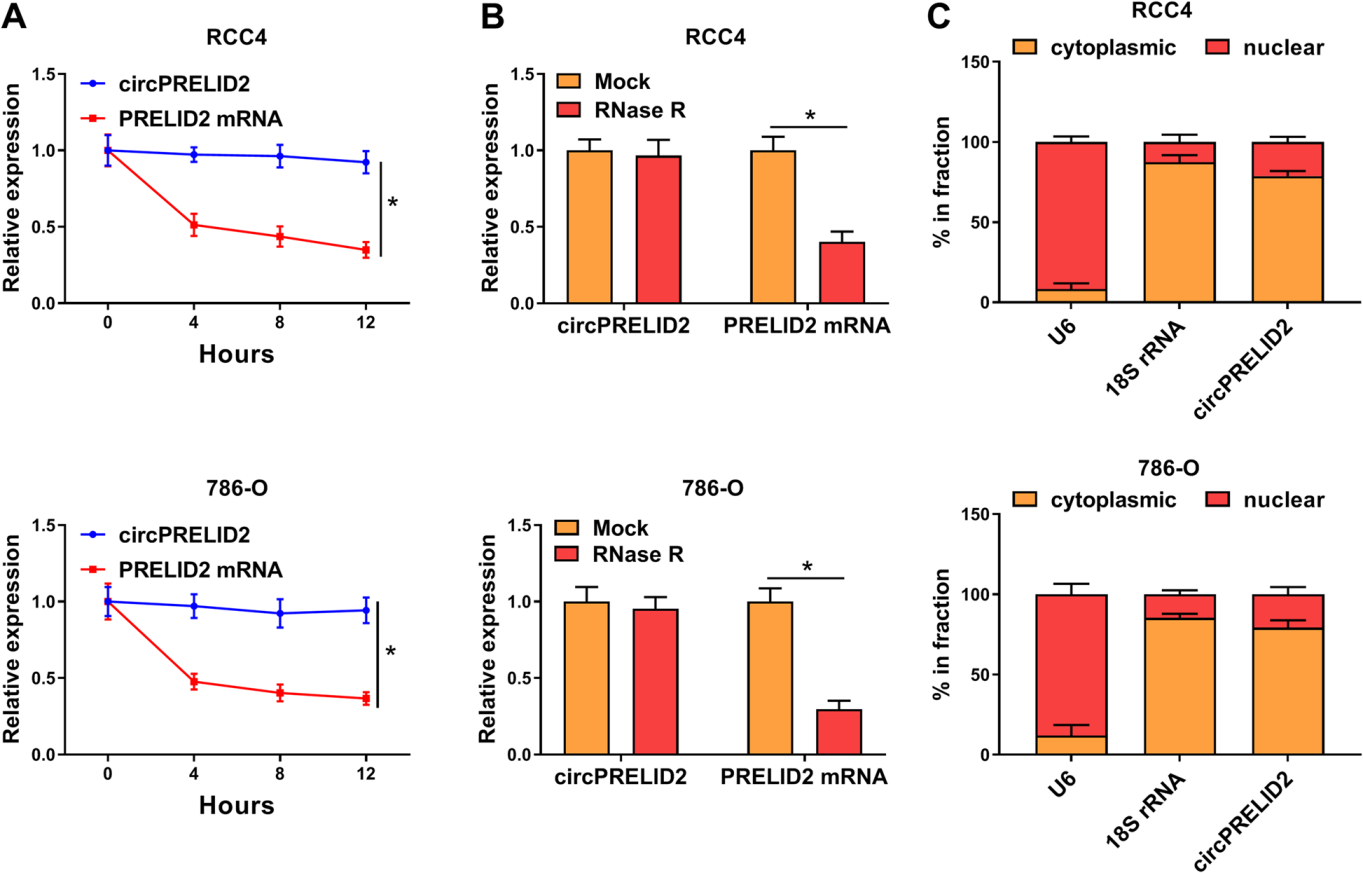
CircPRELID2 is identified in RCC cells. **A** and **B** The expression levels of circPRELID2 by qRT-PCR in both RCC4 and 786-O cells treated with Actinomycin D for 4, 8, 12 and 24 h (**A**), in total RNA digested with RNase R for 15 min (**B**). **C** CircPRELID2 level by qRT-PCR in cytoplasm and nuclear fraction of RCC4 and 786-O cells, with U6 and 18S rRNA as internal controls. *P < 0.05

We then observed the stability of circPRELID2. As a result, Actinomycin D treatment resulted in a significant reduction in PRELID2 mRNA levels (Fig. 2A). However, little decrease was found in the levels of circPRELID2 (Fig. 2A). RNase R treatment demonstrated that circPRELID2 was resistant to RNase R digestion (Fig. 2B), implying that circPRELID2 was relative stable in both RCC4 and 786-O cell lines. Moreover, circPRELID2 was mainly located in the cytoplasm of RCC4 and 786-O cells (Fig. 2C).

### CircPRELID2 silencing suppresses RCC cell malignant behaviors *in vitro *and* in vivo*

**Fig. 3:**
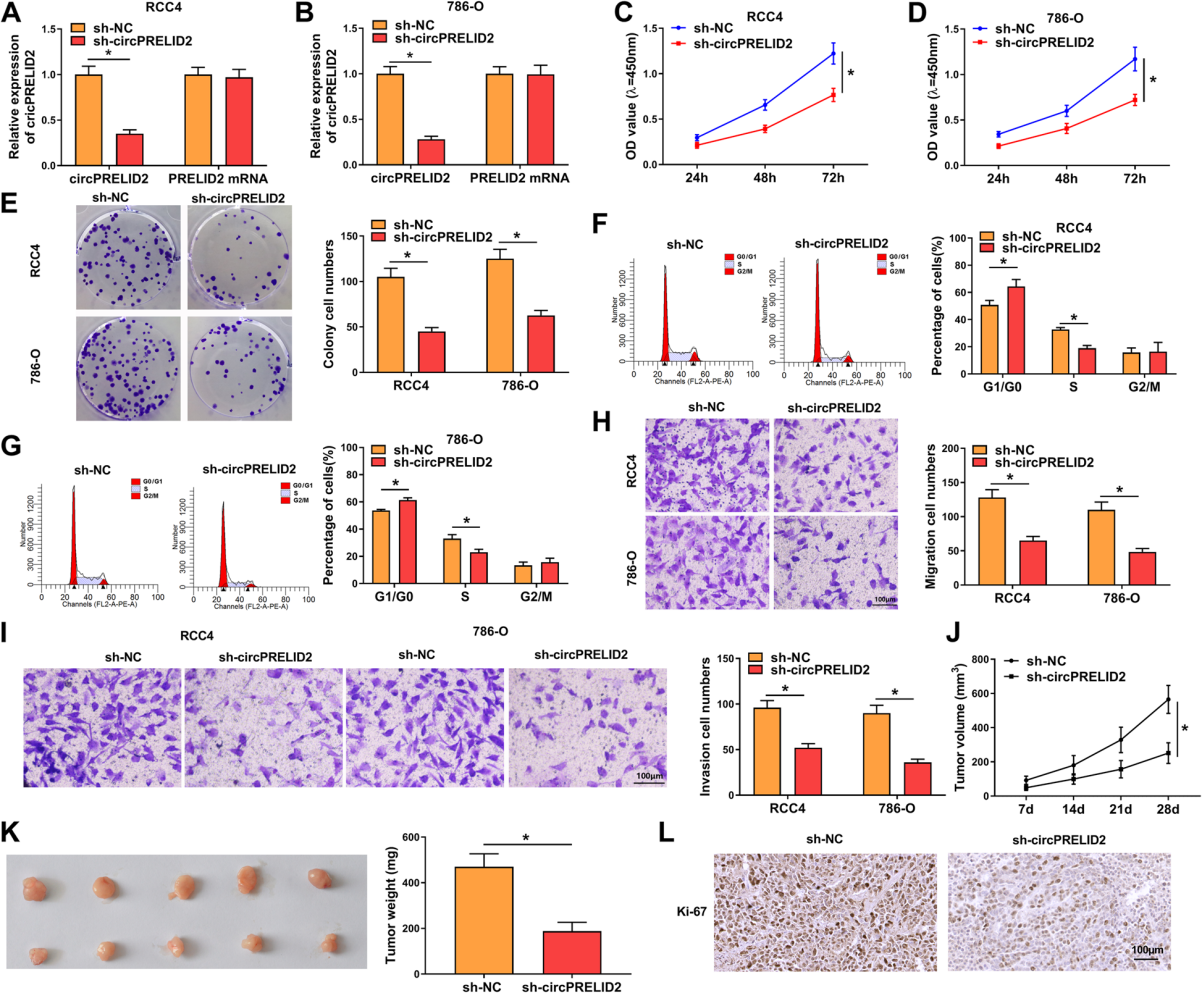
CircPRELID2 silencing represses RCC cell malignant phenotypes in vitro and in vivo. **A**-**I** RCC4 and 786-O cells were transfected with sh-circPRELID2 or sh-NC, followed by the determination of circPRELID2 level by qRT-PCR after 48 h transfection (**A** and **B**), cell proliferation by CCK-8 assay after 24, 48 and 72 h transfection (**C** and **D**), cell colony formation using a standard colony formation assay (**E**), cell cycle progression by flow cytometry after 48 h transfection (**F** and **G**), cell migration and invasion by transwell assay after 24 h transfection (**H** and **I**). **J**-**L** sh-NC- or sh-circPRELID2-transduced RCC4 cells were injected into nude mice (5 mice each group). After 28 days, xenograft tumors were collected. **J** Growth curve of the xenografts. **K** Weight of the xenografts. **L** Immunohistochemistry for Ki-67 staining in the xenografts. *P < 0.05

To investigate the function of circPRELID2, “phenocopy” silencing experiments by using a shRNA vector against circPRELID2 (si-circPRELID2) were performed. CircPRELID2 expression was significantly reduced by sh-circPRELID2 in RCC4 and 786-O cells, and PRELID2 mRNA levels did not be influenced by sh-circPRELID2 (Fig. 3A and B). Downregulation of circPRELID2 caused a prominent inhibition in cell proliferation (Fig. 3C and D). In addition, circPRELID2 knockdown resulted in suppressed colony formation (Fig. 3E) and cycle progression (Fig. 3F and G). Additionally, circPRELID2 silencing triggered a repression in cell motility (Fig. 3H) and invasion (Fig. 3I). Importantly, in vivo, circPRELID2 silencing weakened the growth of RCC4 xenograft tumors (Fig. 3J and K). Furthermore, knocking down circPRELID2 led to the reduction of the Ki-67 positive cells in xenograft tumors (Fig. 3L).

### CircPRELID2 silencing suppresses M2-type macrophage polarization in THP1-M0 cells

**Fig. 4:**
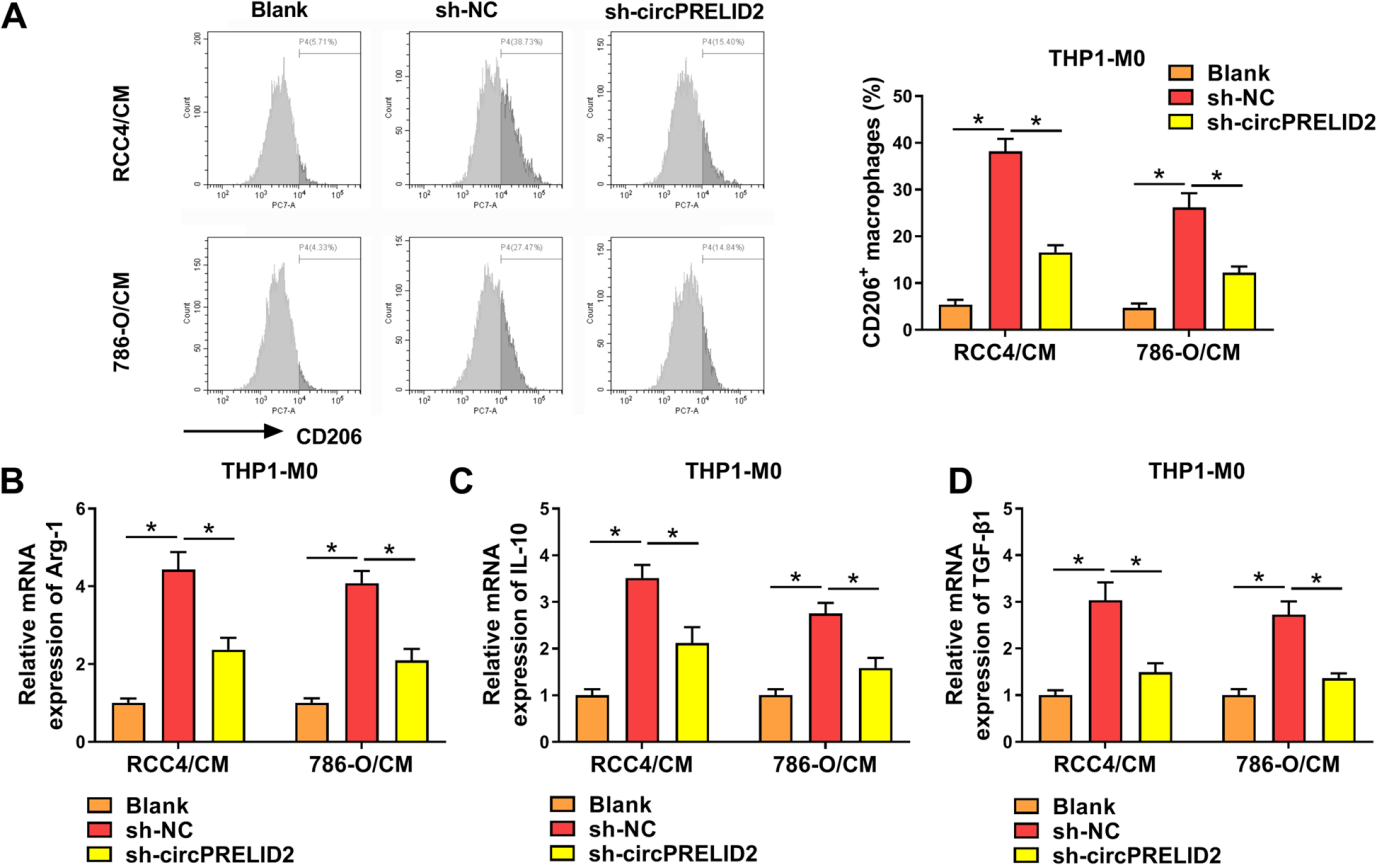
CircPRELID2 silencing suppresses M2-type macrophage polarization in THP1-M0 cells. **A**-**D** The culture media were collected and used to treat THP-1 monocytic leukemia cells co-treated with 100 ng/mL PMA (Sigma-Aldrich) for macrophage (THP1-M0) induction. After 24 h, the cells were assayed. **A** Flow cytometry for the CD206 + macrophages. **B**-**D** qRT-PCR for mRNA expression of Arg-1, IL-10 and TGF-β1. *P < 0.05

To further elucidate the biological function of circPRELID2, we evaluated its effect on M2-type macrophage polarization because M2-polarized macrophages are related to tumor-associated macrophages and exert pro-tumorigenic activity in human cancer [19]. Flow cytometry data revealed that knocking down circPRELID2 caused a striking reduction in the number of the CD206^+^ macrophages (Fig. 4A). Additionally, we assessed the expression levels of the markers (Arg-1, IL-10 and TGF-β1) of M2-polarized macrophages. PMA-induced THP-1 macrophages (THP1-M0) showed lower mRNA levels of Arg-1, IL-10 and TGF-β1 than controls (Fig. 4B-D).

### CircPRELID2 sequesters miR-22-3p and increases ETV1 through miR-22-3p

**Fig. 5:**
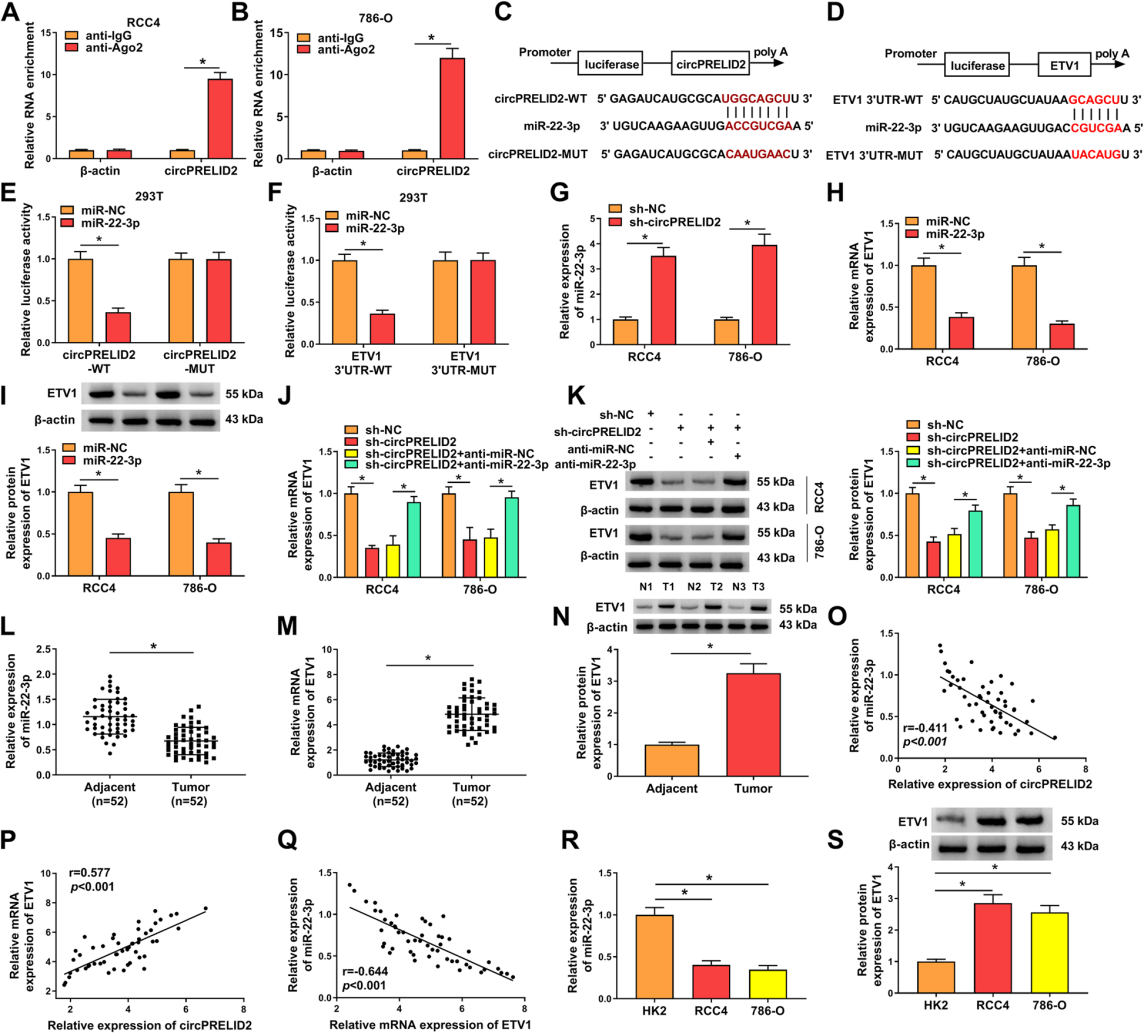
CircPRELID2 acts as a miR-22-3p sponge to regulate ETV1 expression. **A** and **B** Cellular lysates of RCC4 and 786-O cells were incubated with anti-Ago2 or anti-IgG antibody, and then circPRELID2 level was detected by qRT-PCR. **C** Schematic model of the miR-22-3p-binding sites within circPRELID2 and the mutation in the seed sites. **D** Schematic of illuminating the miR-22-3p-binding sites within the 3’-UTR of ETV1 and mutated miR-22-3p-binding sites. **E** CircPRELID2 wide-type or mutant-type reporter construct (circPRELID2-WT or circPRELID2-MUT) was introduced into 293 T cells together with miR-22-3p mimic or miR-NC mimic, and then luciferase activity was assessed. **F** Relative luciferase activity in the cells co-transfected with ETV1 3’-UTR wild-type reporter (ETV1 3’-UTR-WT) or mutant-type reporter (ETV1 3’-UTR-MUT) and miR-NC mimic or miR-22-3p mimic. **G** MiR-22-3p expression was determined by qRT-PCR in RCC4 and 786-O cells transfected with sh-NC or sh-circPRELID2. **H** and **I** qRT-PCR and immunoblotting for ETV1 mRNA and protein levels in the cells transfected with miR-NC mimic or miR-22-3p mimic. **J** and **K** ETV1 expression in cells introduced with si-NC, si-circPRELID2, si-circPRELID2 + anti-miR-NC or si-circPRELID2 + anti-miR-22-3p. **L** MiR-22-3p expression was detected in 52 pairs of clinical RCC tissues and matched healthy tissues. (M and N) ETV1 expression in RCC tissues and matched healthy tissues. **O**-**Q** Expression correlations among miR-22-3p, circPRELID2, ETV1 were tested in RCC tissues using Spearman test. **R** MiR-22-3p expression was detected in HK2, RCC4 and 786-O cells. **S** ETV1 protein expression in HK2, RCC4 and 786-O cells. *P < 0.05

We next explored the ceRNA molecular process underlying the function of circPRELID2. Using the anti-Ago2 antibody, RIP experiments showed that circPRELID2 was strongly enriched, suggesting the endogenous interaction between circPRELID2 and miRNAs (Fig. 5A and B). MiR-22-3p was predicted as a potential miRNA that bind to circPRELID2 using circBank (Fig. 5C). We also used several computational methods (TargetScan, miRWalk, StarBase and miRcode) to help identify the potential targets of miR-22-3p and found that ETV1, a critical regulator in the progression of RCC [20], contained a putative binding site for miR-22-3p (Fig. 5D). To determine whether circPRELID2 functioned as a sponge of miR-22-3p and whether ETV1 was a target of miR-22-3p, we cloned the circPRELID2 segment or ETV1 3’-UTR into the pmirGLO vector and mutated the seed sites in the reporter constructs (Fig. 5C and D). Introduction of miR-22-3p mimic weakened the luciferase activity of wild-type reporter construct (Fig. 5E and F). However, the reduction of miR-22-3p in luciferase was completely abolished by mutant-type constructs (Fig. 5E and F). Moreover, miR-22-3p expression was enhanced by circPRELID2 silencing in both RCC4 and 786-O cell lines (Fig. 5G). ETV1 expression was markedly decreased by miR-22-3p mimic (Fig. 5H and I). More interestingly, these data also demonstrated that ETV1 expression was down-regulated by circPRELID2 silencing in the two cell lines, and this effect was highly reversed by miR-22-3p reduction (Fig. 5J and K). In RCC tissues, miR-22-3p expression was prominently down-regulated, and ETV1 levels were enhanced (Fig. 5L-N). In addition, in RCC samples, circPRELID2 expression was negatively correlated with miR-22-3p and positively correlated with ETV1 level, and miR-22-3p expression was inversely correlated with ETV1 level (Fig. 5O-Q). Consistent with RCC tumor tissues, miR-22-3p expression was down-regulated and ETV1 levels were augmented in RCC cells compared with normal HK2 cells (Fig. 5R and S).

### The miR-22-3p/ETV1 axis is involved in the inhibitory impact of circPRELID2 silencing on RCC cell malignant phenotypes

**Fig. 6:**
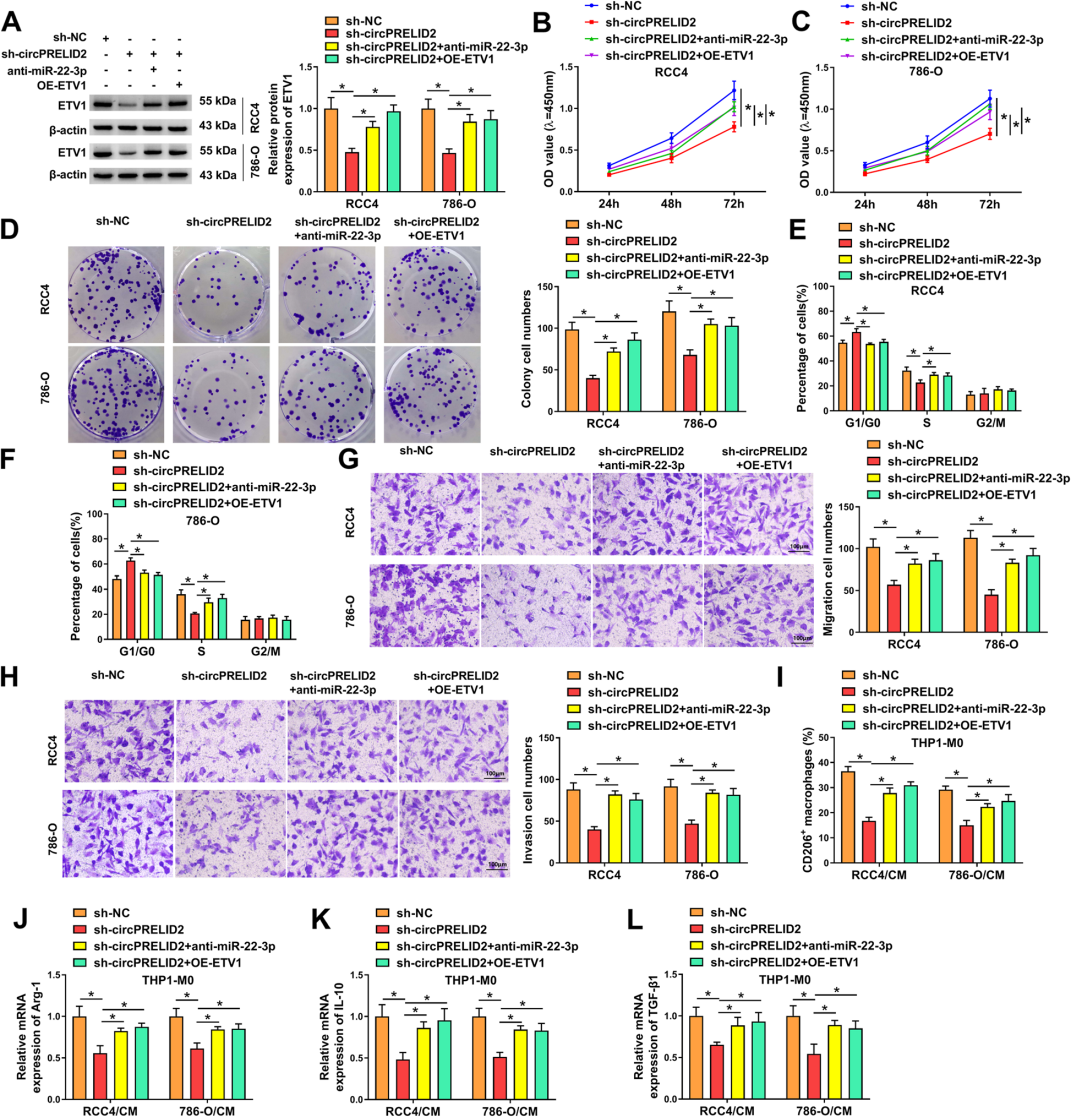
CircPRELID2 silencing suppresses RCC cell malignant phenotypes by the miR-22-3p/ETV1 axis. **A**-**H** RCC4 and 786-O cells were transfected with sh-NC, sh-circPRELID2, sh-circPRELID2 + anti-miR-22-3p or sh-circPRELID2 + OE-ETV1. **A** Immunoblotting for ETV1 protein expression in the transfected cells. **B** and **C** Cell proliferation was assessed. **D** Cell colony formation using a standard colony formation assay. **E** and **F** Cell cycle progression by flow cytometry after 48 h transfection. **G** and **H** cell migration and invasion by transwell assay after 24 h transfection. **I**-**L** The culture media of transfected cells were collected and used to treat THP-1 monocytic leukemia cells co-treated with 100 ng/mL PMA (Sigma-Aldrich) for macrophage (THP1-M0) induction. After 24 h, the cells were assayed. **I** Flow cytometry for the CD206 + macrophages. **J**-**L** qRT-PCR for mRNA expression of Arg-1, IL-10 and TGF-β1. *P < 0.05

In RCC4 and 786-O cells, downregulation of miR-22-3p by anti-miR-22-3p introduction or re-expression of ETV1 by OE-ETV1 transfection abated sh-circPRELID2-driven ETV1 reduction (Fig. 6A). Importantly, downregulation of miR-22-3p or restoration of ETV1 significantly abrogated sh-circPRELID2-imposed cell growth suppression (Fig. 6B-F), motility enhancement (Fig. 6G) and invasion promotion (Fig. 6H). Furthermore, downregulation of miR-22-3p or restoration of ETV1 reversed sh-circPRELID2-mediated reduction of the CD206^+^ macrophages (Fig. 6I) and suppression of Arg-1, IL-10 and TGF-β1 expression (Fig. 6J-L).

## Discussion

A number of circRNAs actively participate in RCC carcinogenesis through miRNA sequestration, repressing their ability to target mRNAs [21]. CircPRELID2 can contribute to breast tumorigenesis via miR-1299 and miR-1236-3p competition [13, 14]. Given the dysregulation of circPRELID2 in RCC, we wanted to define the influence of circPRELID2 on RCC development.

Our data demonstrate that circPRELID2 levels are up-regulated in RCC, and high circPRELID2 expression is associated with RCC malignant process, implying the potential of circPRELID2 as a biomarker for RCC diagnosis and progression. As confirmed for other circRNAs [18, 22], circPRELID2 is unusually stable in RCC cells. Ki-67 and PCNA are the indicators of tumor cell proliferation [23, 24]. In the current study, we uncover, for the first time, that circPRELID2 silencing hampers RCC cell growth, motility and invasion. Importantly, we demonstrate that circPRELID2 silencing can suppress M2-type macrophage polarization in THP1-M0 cells. Thus, inhibiting circPRELID2 may be a promising method to treat RCC. Additionally, similar to several other circRNAs [25, 26], we confirm that circPRELID2 is mainly localized in the cytoplasm of RCC cells. Using the anti-Ago2 antibody, circPRELID2 was found to be significantly enriched in the RISC, offering the possibility of the endogenous circPRELID2 interplay with miRNAs in RCC.

MiR-22-3p is intriguing for our study due to its critical involvement in pancreatic cancer, cervical squamous carcinoma, and hepatocellular carcinoma [27–29]. Moreover, miR-22-3p is reported to participate in circ-ITCH-mediated regulation in the development of papillary thyroid cancer [30]. Former work also uncovers that miR-22-3p can function as a cancer repressor in RCC [15]. Using several bioinformatics methods, we conducted a detailed analysis for miR-22-3p targets. We selected ETV1 for the further exploration because it is down-regulated by miR-22-3p and is correlated with RCC pathogenesis [20]. Previous documents report that up-regulated ETV1 enhances tumor aggressiveness in pancreatic cancer, prostate cancer and gastrointestinal stromal tumor [31–33]. Several miRNAs, including miR-129-5p and miR-17-5p, are proved to mitigate tumor development by targeting ETV1 [34, 35]. Here, we validate, for the first time, that circPRELID2 sequesters miR-22-3p. Furthermore, our data illuminate that the inhibitory influence of circPRELID2 depletion on RCC cell malignant behaviors is mediated by the miR-22-3p/ETV1 axis. With these findings, the sh-circPRELID2 plasmid may be a promising anti-RCC agent that hinders RCC malignant progression and suppresses M2-type macrophage polarization. We envision that the circPRELID2 inhibitors are a starting point for development of circRNA-based molecular therapies against RCC.

Although our findings uncover the implication of the circPRELID2/miR-22-3p/ETV1 axis in RCC progression in vitro, its regulation in vivo is lacking in the current work by using various RCC animal models, which is a major limitation in our study. Our data also suggest that circPRELID2 may be a biomarker for RCC diagnosis and progression, which needs to be further proved because of the small cohort of RCC patients used in this report. Additionally, our research findings by using two RCC cell lines are inadequate, and more RCC cell lines should be used for demonstration of this novel mechanism.

In conclusion, to our knowledge, this is the first study of the action of circPRELID2 in RCC. Depletion of circPRELID2 hampers RCC malignant progression through the miR-22-3p/ETV1 cascade. This study highlights a new therapeutic target, the circPRELID2/miR-22-3p/ETV1 axis, for RCC treatment.

### Supplementary Information

**Additional file 1:** **Supplement Table 1.** Primers for PCR.

**Additional file 2: **The original blot images.

## Data Availability

The anonymised raw datasets used and/or analysed during the current study are available from the corresponding author on reasonable request.
