# Supplementary material for: CircPRELID2 functions as a promoter of renal cell carcinoma through the miR-22-3p/ETV1 cascade
Source: BMC Urol. 2024 May 10;24:104. doi: 10.1186/s12894-024-01490-z (PMC11088145; doi:10.1186/s12894-024-01490-z)
Supplement: Supplementary file 1 — Additional file 1: Supplement Table 1. Primers for PCR. [file 12894_2024_1490_MOESM1_ESM.docx]

**Supplement Table 1. Primers for PCR.**

| Primers for PCR (5’-3’) | | |
| --- | --- | --- |
| circPRELID2 | Forward | GAAACATGGCCATACGGAGT |
|  | Reverse | CGCAGGGTGCTATCCTAGAG |
| PRELID2 mRNA | Forward | TGTCAGAACGTGGTTCCAGA |
|  | Reverse | CCATACTTTCCCGGAAGACA |
| 18S rRNA | Forward | GCGAAAGCATTTGCCAAGAA |
|  | Reverse | GATCCTTCCGCAGGTTCACCT |
| miR-22-3p | Forward | AAGCTGCCAGTTGAAGAACTGT |
|  | Reverse | GGCCAACCGCGAGAAGATGTTTTTTTTT |
| AK2 | Forward | TACTCAGCGGCCGCATGGCTCCAGCAGCTCCTGCA |
|  | Reverse | TACTCACTCGAGTTACTGGGCCGCCCTTTGCTT |
| SCYL3 | Forward | ATATAATAATAAAGAGACAAAAGAGGC |
|  | Reverse | TACTCTTTATTAGATGCATTACTTTCA |
| CAPN1 | Forward | GAAGGCAACGAGTTCTGGAG |
|  | Reverse | GGTCCACGTTGTTCCACTCT |
| NFYA | Forward | CCGGAATTCATG GAGCAGTATACAGCA |
|  | Reverse | CCGCTCGAGTTAGGACACTCGGATGA |
| ETV1 | Forward | ATGGATGGATTTTATGACCAG |
|  | Reverse | TTAATACACGTAGCCTTCGTTG |
| SLC7A2 | Forward | GGTGCTGAGGTGGGAGGAT |
|  | Reverse | AGAGGAGGGAAGGCAAGG |
| β-actin | Forward | GCACCACACCTTCTACAATG |
|  | Reverse | TGCTTGCTGATCCACATCTG |
| U6 | Forward | CTCGCTTCGGCAGCACA |
|  | Reverse | AACGCTTCACGAATTTGCGT |
