# Supplementary material for: CircPRELID2 functions as a promoter of renal cell carcinoma through the miR-22-3p/ETV1 cascade
Source: BMC Urol. 2024 May 10;24:104. doi: 10.1186/s12894-024-01490-z (PMC11088145; doi:10.1186/s12894-024-01490-z)
Supplement: Supplementary file 2 — Additional file 2: The original blot images. [file 12894_2024_1490_MOESM2_ESM.pdf]

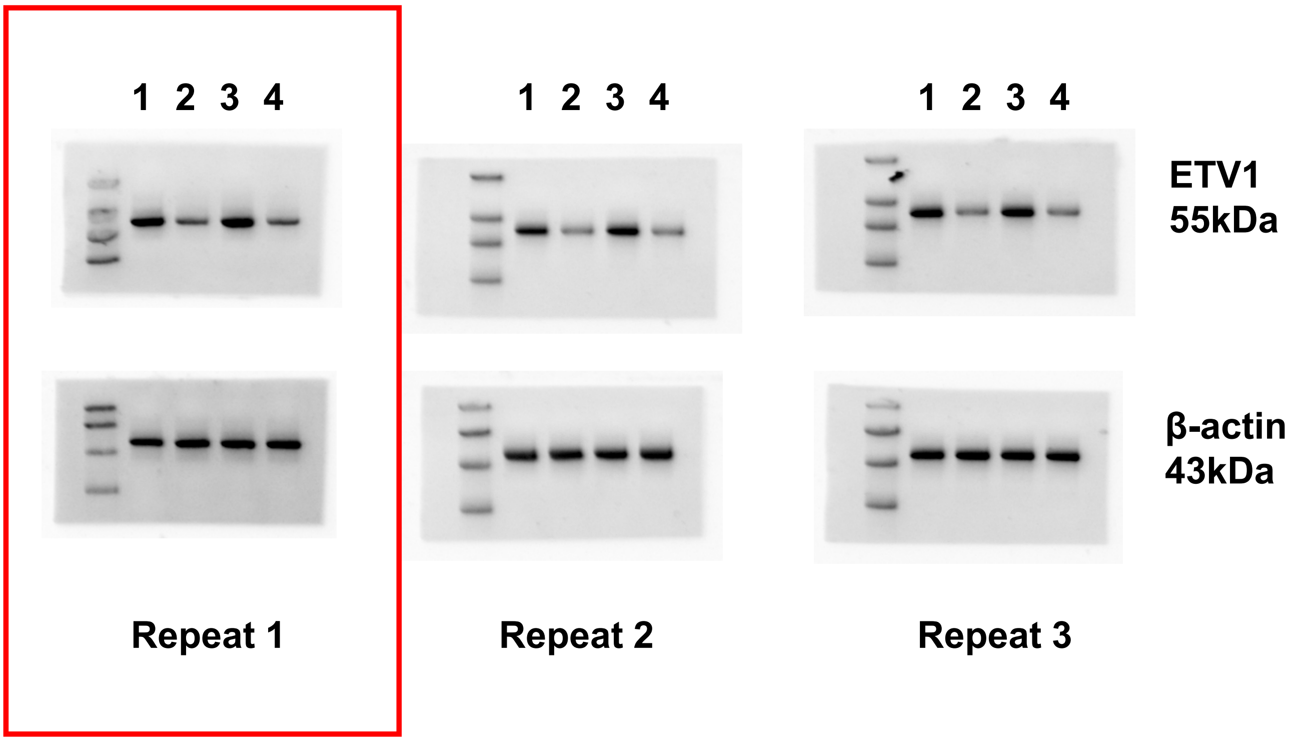

Fig 5I

1 RCC4 miR-NC

2 RCC4 miR-22-3p

3 786-O miR-NC

4 786-O miR-22-3p

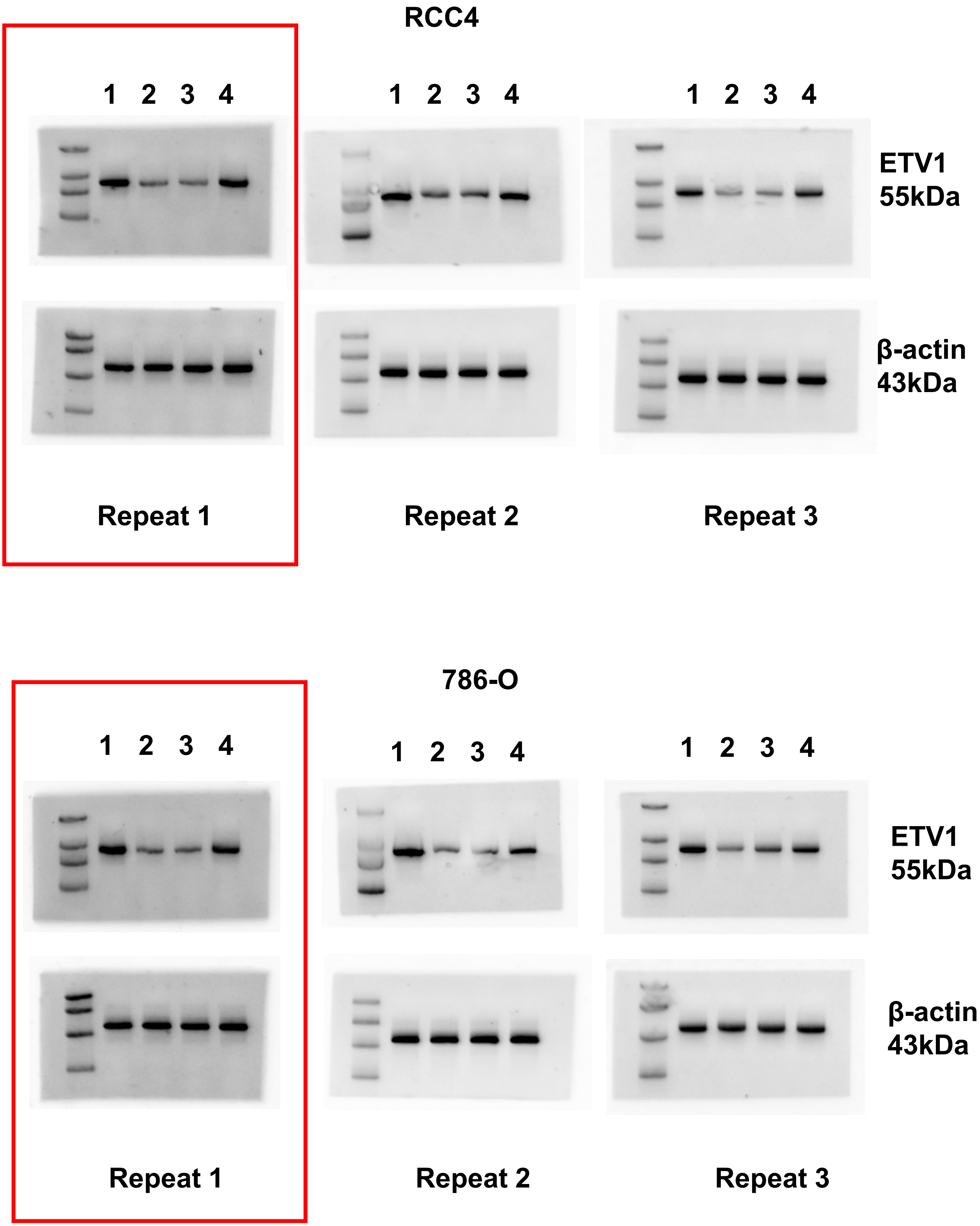

Fig 5K

1 sh-NC

2 sh-circPRELID2

3 sh-circPRELID2+anti-miR-NC

4 sh-circPRELID2+anti-miR-22-3p

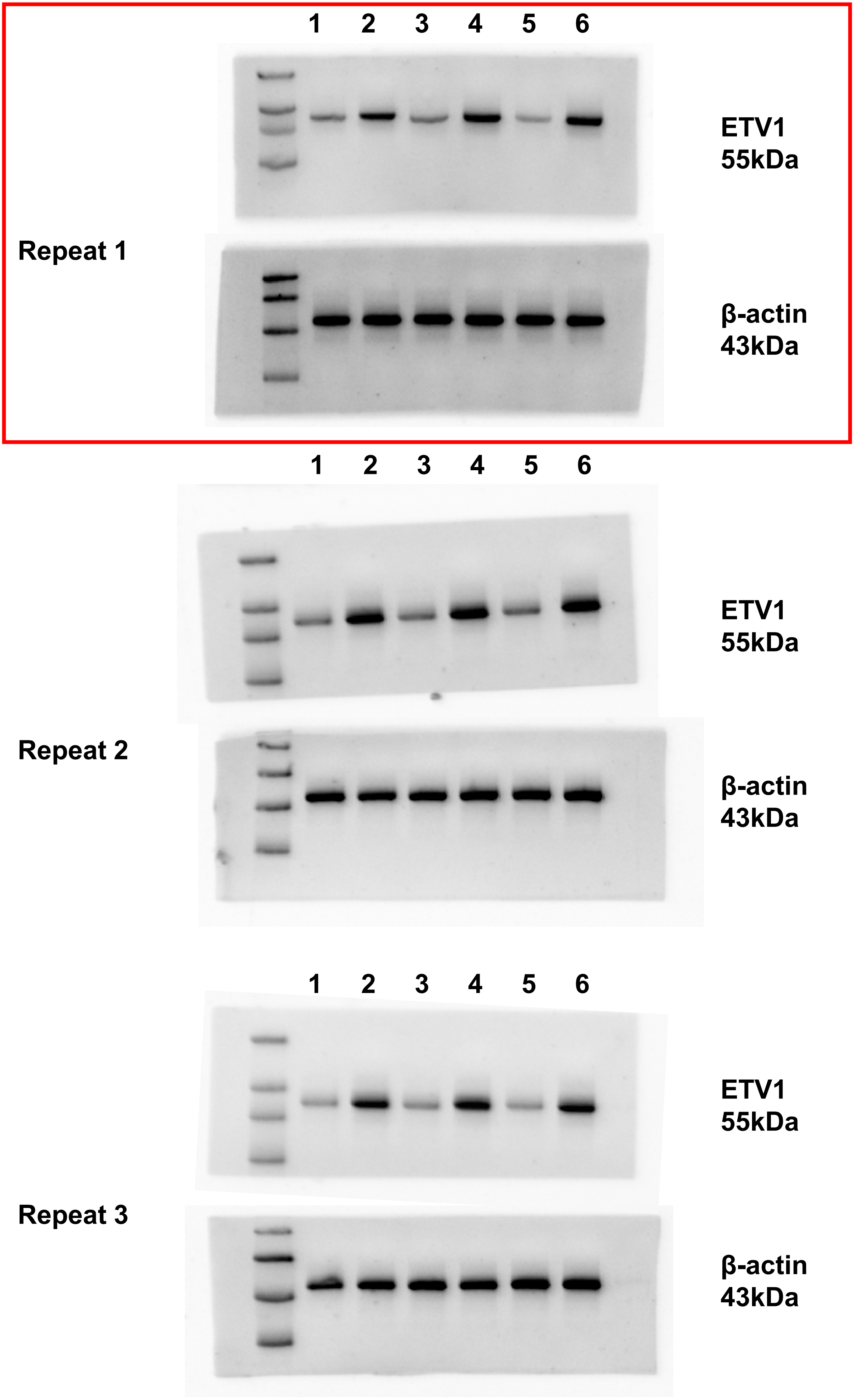

1 N1 3 N2 5 N3
2 T1 4 T2 6 T3

Fig 5N

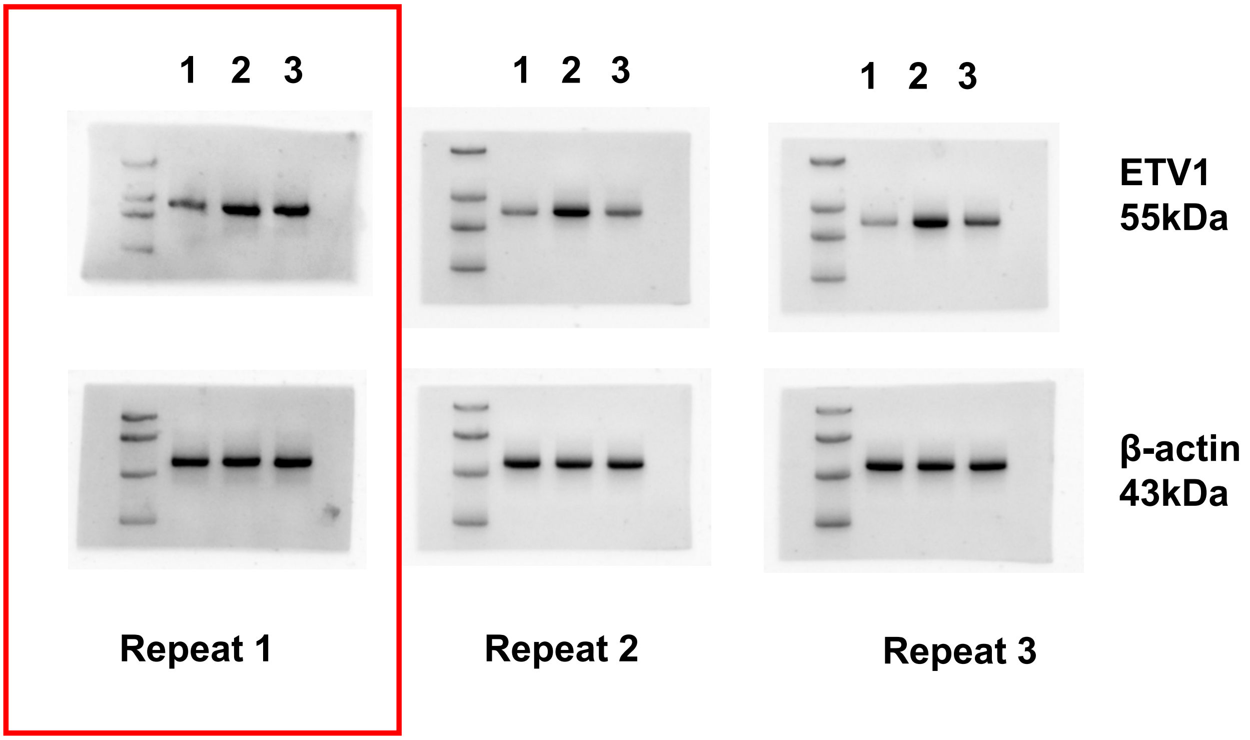

Fig 5S 1 HK2

2 RCC4

3 786-O

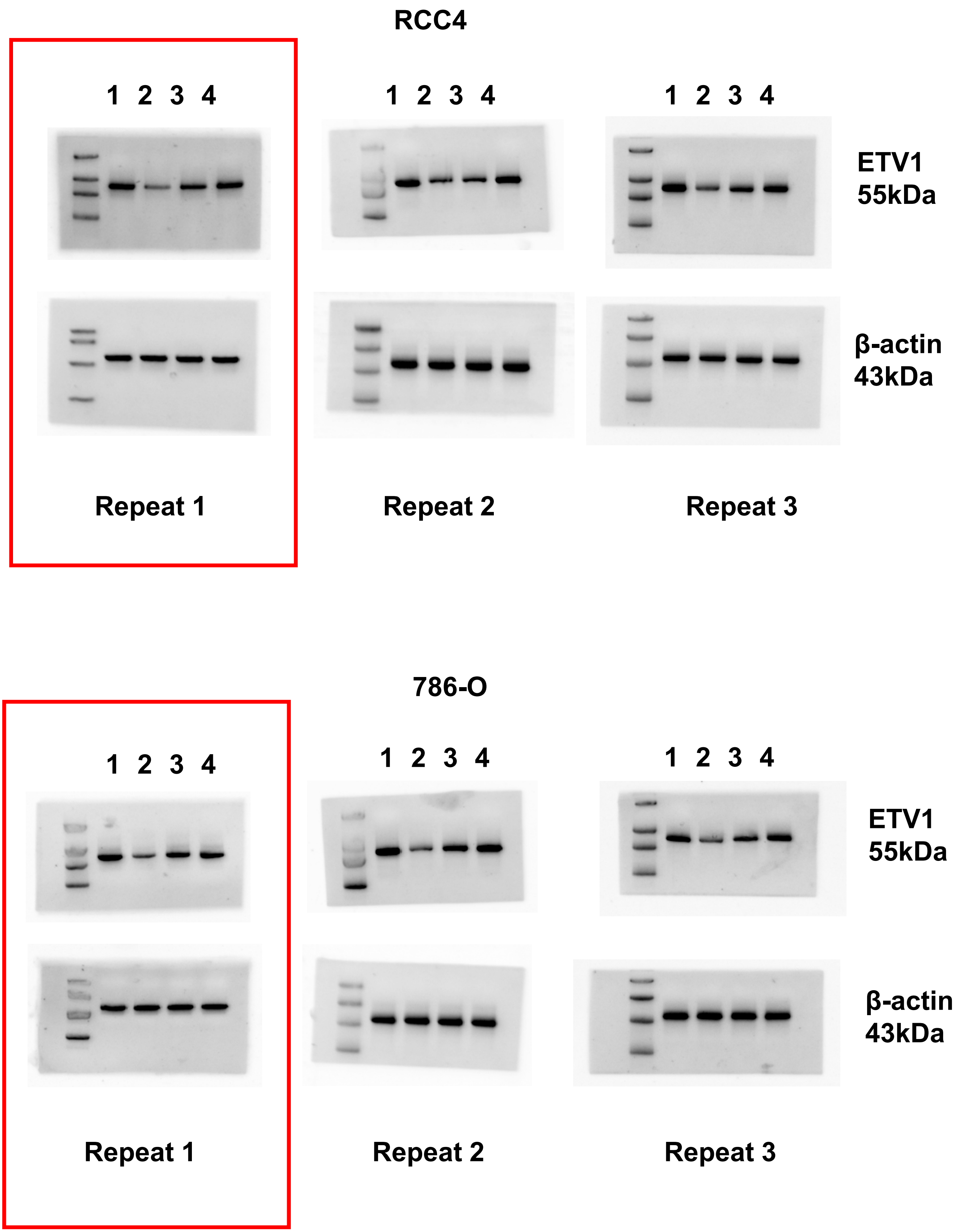

Fig 6A

1 sh-NC

2 sh-circPRELID2

3 sh-circPRELID2+anti-miR-22-3p

4 sh-circPRELID2+OE-ETV1

Protein Marker

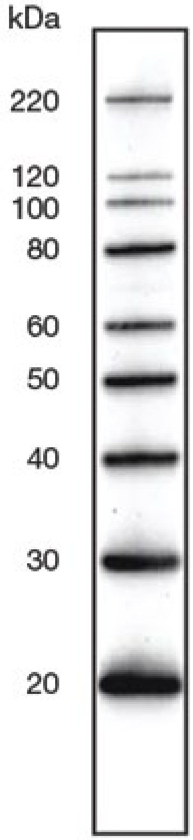

- **The samples derive from the same experiment and that gels/blots were processed in parallel.**
